# Fe^3+^/Mn^2+^ (Oxy)Hydroxide Nanoparticles Loaded onto Muscovite/Zeolite Composites (Powder, Pellets and Monoliths): Phosphate Carriers from Urban Wastewater to Soil

**DOI:** 10.3390/nano12213848

**Published:** 2022-10-31

**Authors:** Diana Guaya, Luz Maza, Adriana Angamarca, Eda Mendoza, Luis García, César Valderrama, José Luis Cortina

**Affiliations:** 1 Department of Chemistry, Universidad Técnica Particular de Loja, Loja 100107, Ecuador; 2 Department of Chemical Engineering, Polytechnic University of Catalonia–BarcelonaTech (UPC), 08019 Barcelona, Spain; 3 Barcelona Research Center for Multiscale Science and Engineering, 08930 Barcelona, Spain

**Keywords:** phosphate, adsorption, kinetic, equilibrium, batch, fixed-bed column

## Abstract

The development of an efficient adsorbent is required in tertiary wastewater treatment stages to reduce the phosphate–phosphorous content within regulatory levels (1 mg L^−1^ total phosphorous). In this study, a natural muscovite was used for the preparation of muscovite/zeolite composites and the incorporation of Fe^3+^/Mn^2+^ (oxy)hydroxide nanoparticles for the recovery of phosphate from synthetic wastewater. The raw muscovite MC and the obtained muscovite/sodalite composite LMC were used in the powder form for the phosphate adsorption in batch mode. A muscovite/analcime composite was obtained in the pellets PLMCT_3_ and monolith SLMCT_2_ forms for the evaluation in fixed-bed mode for continuous operation. The effect of pH, equilibrium and kinetic parameters on phosphate adsorption and its further reuse in sorption–desorption cycles were determined. The characterization of the adsorbents determined the Fe^3+^ and Mn^2+^ incorporation into the muscovite/zeolite composite’s structure followed the occupancy of the extra-framework octahedral and in the framework tetrahedral sites, precipitation and inner sphere complexation. The adsorbents used in this study (MC, LMC, PLMCT_3_ and SLMCT_2_) were effective for the phosphate recovery without pH adjustment requirements for real treated wastewater. Physical (e.g., electrostatic attraction) and chemical (complexation reactions) adsorption occurred between the protonated Fe^3+^/Mn^2+^ (oxy)hydroxy groups and phosphate anions. Higher ratios of adsorption capacities were obtained by powder materials (MC and LMC) than the pellets and monoliths forms (PLMCT_3_ and SLMCT_2_). The equilibrium adsorption of phosphate was reached within 30 min for powder forms (MC and LMC) and 150 min for pellets and monoliths forms (PLMCT_3_ and SLMCT_2_); because the phosphate adsorption was governed by the diffusion through the internal pores. The adsorbents used in this study can be applied for phosphate recovery from wastewater treatment plants in batch or fixed-bed mode with limited reusability. However, they have the edge of environmentally friendly final disposal being promissory materials for soil amendment applications.

## 1. Introduction

Phosphorous (P) is an essential element for human life, such is the case of global food production. The ever-increasing population worldwide has promoted a potential demand of fertilizer products because soil fertility is crucial for agriculture [1]. However, the limited availability of phosphorous resources (e.g., phosphate rocks) is well known. In order to meet the agricultural demand, the consumption of phosphate rock raises an average of 3% per year. However, the rising demand for fertilizers implies a concern about the phosphate rock supply worldwide whose depletion is estimated in the next century [2]. The potential responses to phosphorous scarcity may comprise the cost increase, the efficient use of fertilizers and the phosphorous recovery and re-use [3].

In fact, worldwide the 5Rs strategy (Realign P inputs in agriculture, Reduce P losses in the hydrosphere, Recycle P in bio-resources, Recover P from waste and Redefine the food system) is being adopted to overcome the increase in the price of fertilizers and food [2]. The European Union promotes the “large scale fertilizer production in the EU from domestic organic or secondary raw materials” [4]. Thus, the use of urban wastewater is imperative, being the most important secondary source of phosphorous and containing the phosphate–phosphorous from detergents, household wastes, agricultural runoff and farming wastes [5]. The re-use of phosphorus from wastewater contributes simultaneously with the accomplishment of two objectives for sustainable development, established by the United Nations Organisation: Goal 2: “end hunger, achieve food security and improved nutrition and promote sustainable agriculture” and Goal 6: “ensure access to water and sanitation for all” [6]. Thus, the phosphate–phosphorous recovery from wastewater could be a viable solution for the phosphorous scarcity, as well as become an alternative of treatment for the reduction in phosphate–phosphorous contents in wastewater.

With this background, the orthophosphate anionic form (i.e., H_2_PO_4_ and HPO_4_^2−^) from wastewater treatment plants (WWTP) together with ammonium (N-NH_4_^+^) and potassium (K^+^) cations are the main cause of eutrophication of natural water bodies. The accumulation of orthophosphate provides optimal conditions for fast growing of aquatic organisms being a serious environmental problem [7]. The excessive algae growing causes turbidity, hypoxia, malodour and toxins excretion in waters becoming a public health problem [8]. New promising technologies are studied for phosphate–phosphorous reduction within the regulatory levels (1 mg L^−1^ total phosphorous). Adsorption has been reported as promising technology for phosphate recovery due to the low cost, easy operation and high efficiency and selectivity [9]. Many adsorbents have been used for this purpose, i.e., clays [5], natural zeolites [10], polymeric exchangers [11] and LDHs [12]. However, some advantages and disadvantages are associated to the phosphate adsorbents from some technical and environmental points of view.

It is advantageous the use of polymeric exchangers due to the high mechanical strength, selectivity, regeneration and easy operation in continuous phosphate adsorption mode [13]. However, the main restriction is their final disposal which is not environmentally friendly. Thereby, the use minerals (e.g., aluminosilicates) are preferred by the opportunity of final soil amendment application because they do not represent a risk by the release of toxic pollutants [14]. Conventionally, the use of layered aluminosilicates minerals is reported as an efficient method for water remediation applications. Some new inorganic composites between clays and zeolites (clay/zeolite composites) have been developed with potentiate properties as super-adsorbents for phosphate removal [15]. The use of inorganic materials include kaolinite, attapulgite, montmorillonite and mica; which improve the properties of super-adsorbents. The use of muscovite-mica has been reported for the development of super-absorbents of low-cost, heat-durability, alkali and salt resistance [16]. Some limitations also are associated to the use of minerals due to the lack of chemical and mineralogical reproducibility, and the implementation of continuous mode operation systems is not viable due to the particle size problems.

Within the mica group of clay minerals, muscovite is a phyllosilicate natural mineral saturated with potassium ions in the interlayer. Muscovite in the raw form develops excellent adsorption properties for anions and cations [17]. However, some innovative composites based on muscovite have been developed for adsorption or catalytic purposes for environmental applications (e.g., muscovite/sodalite [17], muscovite/phillipsite [15], muscovite/TiO_2_ [18], Mn/Mg-Al/LDHs [19], carbon materials-GDE [20], L-lysine modified montmorillonite [21] and functionalized LDH [19]). The well-known negative charge of clays and zeolites are important for cation adsorption properties, but promote low phosphate adsorption capacities. Thus, the potentiation of anion adsorption properties of the muscovite/zeolite composites are performed by the incorporation of metal ions (e.g., iron, aluminium and manganese), due to the high affinity between phosphate oxyanion and metal (oxy)hydroxide [1].

In this this study the obtaining of Fe^3+^/Mn^2+^ (oxy)hydroxide nanoparticles loaded onto muscovite/zeolite composites were performed as novel materials, since we have not found previous reports with detailed experimental information. The metal (oxy)hydroxide nanoparticles have been widely reported to be excellent removers of phosphate by means of surface complexes. The adsorption on metal (oxy)hydroxide nanoparticles are dependent of the crystallinity, specific area and the concentration of OH groups on the surface of the material [22]. Some nanoscale materials have been used in different science fields, such as in energy they improve the surface reactivity of active sites [23]. Particularly in the environmental field, the nanoparticles highly improve the adsorption efficiency of pollutants due to the high specific surface area [24]. However, the major concern of using nanoparticles is the rapidly aggregation effect which can be avoided by supporting them onto templates [14]. The aggregation of nanoparticles can affect their physicochemical properties and the effectiveness of adsorbents. Thus, the formation of metal (oxy)hydroxide nanoparticles over the surface of clay/zeolite composites become a convenient method to control the particle size problem and assure their long-term stability.

The obtaining of powder, pellets and monolith forms of the loaded Fe^3+^/Mn^2+^, muscovite/zeolite composites were performed to validate phosphate removal from synthetic wastewater in batch and continuous mode. Although, powders have high surface area for adsorption purposes, the main limitation is the real application for large wastewater volumes in continuous mode. The pore-clogging, channelling and powder release to effluent are usual problems of working with adsorbents in the powder form. The densification of powder materials into pellets and monoliths forms become a viable alternative to overcome the problems of powders in gas and liquid phase adsorption process [25]. The big challenges of the densification of powder materials are in maintaining or enhancing their original properties; even though they allow the treatment of high flow rates in continuous mode with a reduced pressure drop [26]. The use of monolith forms is reported to be more effective than the use of pellet forms in packed-bed due to the lower pressure drop, but higher cost is associated. However, the effectiveness of the densification forms depend of the preparation and their further characteristics (e.g., surface area, mechanical resistance, mass transfer improvement, pressure drop, reusability and easily commercial application) [27]. It is not clear whether the pellet or the monolith form are effective for phosphate removal. Thus, it is necessary more research to develop a full-scale application for phosphate removal from WWTP.

This study of loaded Fe^3+^/Mn^2+^ (oxy)hydroxide nanoparticles onto muscovite/zeolite composites as phosphate carriers from urban wastewater to soil is focused on phosphate removal mechanisms as well as in their possible regeneration. The Fe^3+^/Mn^2+^ muscovite/zeolite composites (e.g., powder, pellet and monolith forms) are promissory due to the possibility of using these materials in batch and continuous mode, giving them potential for large scale application. The prepared composites also provide high opportunities for soil amendment application and non-polluting characteristics in the final disposal. The loaded Fe^3+^/Mn^2+^ nanoparticles onto muscovite/zeolite composites saturated with phosphate (P) could provide macronutrients (P) and micronutrients (Fe^3+^/Mn^2^) for an agricultural application. Then, the nutrient system P/Fe^3+^/Mn^2+^ could promote soil fertility and improving the plants growth. Moreover, the lack of toxic elements that can be released from Fe^3+^/Mn^2+^ muscovite/zeolite composites makes them environmentally safe for water and soil environments. The objectives of this study are to (i) synthesize the loaded Fe^3+^/Mn^2+^ muscovite/zeolite composites, (ii) obtain and characterize the powder, pellets and monolith forms of the loaded Fe^3+^/Mn^2+^ muscovite/zeolite composites, (iii) verify the influence of sorption parameters on phosphate removal by loaded Fe^3+^/Mn^2+^ muscovite/zeolite composites, (iv) determine the equilibrium and kinetic sorption parameters and (v) validate the regeneration capacity of loaded Fe^3+^/Mn^2+^ muscovite/zeolite composites.

## 2. Materials and Methods

### 2.1. Clay Collection and Pre-Treatment

The raw natural muscovite (MC) used in this study was collected from Loja province at the San Cayetano formation in The Paradise zone (3°57′55.49′′ S, 79°11′45.26′′ W). The raw MC was located in the Loja Miocene Sedimentary Basin located at the Central Andes Cordillera in southern Ecuador. The raw MC was crushed until particles below 200-µm mesh were obtained. The raw MC was washed several times with deionized water and dried for further treatment. The raw MC was thermally activated in an electric furnace at heating rate of 5 °C/min until 600 °C to remove carbonates and organic matter.

### 2.2. Obtaining of Fe^3+^/Mn^2+^ (Oxy)Hydroxide Nanoparticles and Loading onto Muscovite/Sodalite Composite

It was used an adaptation of the method reported by Salam et al. (2021) for the sodalite preparation [17]. Iron and manganese (oxy)hydroxide nanoparticles were incorporated in MC by the co-precipitation method using 30 g of raw MC in 250 mL of a combined solution (0.1 M of FeCl_3_–0.1 M of MnCl_2_) [12]. The slurry was maintained under agitation and reflux (at 150 ± 5 °C) for 4 h at pH 7 using the necessary amount of NaOH solution (1 M). A second treatment stage was performed using the same conditions above-described and refreshing the solution (FeCl_3_–MnCl_2_). The Fe^3+^/Mn^2+^ muscovite/sodalite composite (LMC) sample was washed with deionized water to remove the excess of NaOH and iron–manganese chloride. The LMC was dried at 80 °C for 24 h for further use and storage.

### 2.3. Obtaining of Fe^3+^/Mn^2+^ (Oxy)Hydroxide Nanoparticles Loaded onto Muscovite/Analcime Ceramic Composites (Monoliths and Pellets)

**Figure 1:**
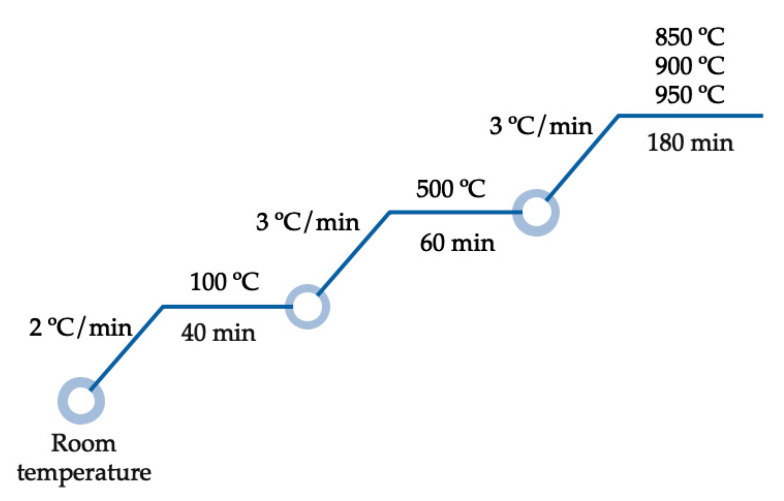
Experimental conditions for the preparation of composites: monoliths (SLMC) and pellets (PLMC).

A homogenous solid suspension was obtained by stirring 45% of LMC and 55% of deionized water at 400 rpm for 12 h. For Fe^3+^/Mn^2+^ composite monoliths (SLMC), we obtained cylindric shapes of polyurethane sponges (diameter: 3 ± 0.2 cm × height: 3 ± 0.2 cm). The polyurethane sponges were impregnated with the LMC suspension using a syringe. The monoliths were dried in an oven at 90 °C for 30 min. The procedure was repeated almost four times until the highest mass of suspension was impregnated in the sponge. The composite monoliths (SLMC) were dried at 90 °C for 24 h. The preparation of Fe^3+^/Mn^2+^ composite pellets (PLMC) included the addition of 0.5% of carboxy-methyl-cellulose. A plastic syringe was used to obtain the pellets (diameter: 1.2 ± 0.1 mm x height: 5 ± 0.1 mm). The composite pellets were dried at 90 °C for 24 h. Finally, both composites (SLMC and PLMC) were calcined at T_1_: 850 °C, T_2_: 900 °C and T_3_: 950 °C at a heating rate of 2.5 °C/min for 3 h (Figure 1). Both composites, after being calcined, were treated in a combined solution (0.1 M of FeCl_3_–0.1 M of MnCl_2_) following the co-precipitation process above-described for the Fe^3+^/Mn^2+^ nanoparticles obtaining.

### 2.4. Materials Characterization

The physicochemical characterization of the adsorbents (e.g., MC, LMC, PLMCT_3_ and SLMCT_2_) were performed. A wavelength dispersive X-ray fluorescence spectrometer (Bruker S1, Karlsruhe, Germany) was used to determine the composition of the adsorbent samples. The X-ray diffraction (XRD) patterns were acquired at 25 °C and over an angular range from 4 to 60° of 2θ on a powder X-ray Diffractometer (D8 Advance A25 Bruker, Karlsruhe, Germany) with a Cu Kα anode (λ = 0.1542 nm) operating at 40 kV and 40 mA. The infrared absorption spectra were recorded with a Fourier Transform FTIR spectrometer in the range of 4000–550 cm^−1^ (4100 Jasco, Easton, MD, USA). The morphology surfaces of the adsorbents were studied by a field emission scanning electron microscope (SEM JEOL, Peabody, MA, USA; JSM-7001F, Peabody, MA, USA). The points of zero charge (PZC) of the adsorbents were determined by the pH drift method in the range of pH 2–10 [28], using different ionic strength. The specific surface areas of the adsorbents were determined by the nitrogen gas adsorption single-point method on an automatic sorption analyser using a flow gas containing 30% N_2_-70% He (Micrometrics Chemisorb 2720, Norcross, GA, US).

### 2.5. Phosphate Adsorption Assays in Bath Mode

The phosphate synthetic solution was prepared from a NaH_2_PO_4_.2H_2_O stock solution (1000 mg·L^−^^1^ PO_4_^3^^−^) in deionized water. Samples of the adsorbents (0.25 g MC, LMC and PLMC and 10 ± 0.2 g SLMC) were equilibrated in 25 mL of solution (25 mg·L^−^^1^ PO_4_^3^^−^) at room temperature (21 ± 2 °C). The supernatant was collected after being centrifuged at 5000 rpm and further filtrated (0.45 μm) for the determination of the values of pH and phosphate concentrations at initial and equilibrium state. Phosphate (P) concentration was determined based on the Standard Methods [29]. P-PO_4_^3−^ was determined by the vanadomolybdophosphoric acid colorimetric method (4500-P C) in a Shimadzu UVmini-1240 UVvis spectrophotometer. Overall tests were performed in triplicate and the average values are reported. The specific conditions used for assays will be described properly in each section. The equilibrium adsorption capacity was determined by Equation (1).

(1) $$Q_{e}=\frac{\left(C_{o}-C_{t}\right)\times V}{w}$$

where Q_e_ is the phosphate equilibrium adsorption capacity (mg·g^−^^1^ PO_4_^3^^−^), V is the volume of phosphate solution (L), C_0_ and C_e_ are the initial and equilibrium phosphate concentrations (mg·L^−^^1^ PO_4_^3^^−^), respectively; and w is the mass of the adsorbent material used (g).

#### 2.5.1. Effect of the Calcination Temperature of Composites

Phosphate adsorption was evaluated onto PLMC and SLMC composites prepared at T_1_: 850 °C, T_2_: 900 °C and T_3_: 950 °C; by equilibration at pH 7 ± 0.3 (which is the pH value of a treated urban wastewater) [21]. Moreover, the resistance forces of the composite monoliths were evaluated by supporting some mass weights until the rupture. The product of the mass weight by the gravitational force provided the resistance in newtons. The stabilities of the composite pellets were determined by agitation in the phosphate solutions in terms of disaggregation (ND: not disaggregate, D: partial disaggregate and TD: totally disaggregate).

#### 2.5.2. Effect of the pH

A sample of the selected adsorbent material (MC, LMC, PLMCT_3_ and SLMCT_2_) was used for further assays. The initial pH values of the solutions were adjusted between 2 and 10.

#### 2.5.3. Equilibrium Adsorption Capacity

The equilibrium adsorption capacity was evaluated using solutions containing 10–2000 mg·L^−^^1^ PO_4_^3^^−^ at pH 7 ± 0.3 (which is the pH value of a treated urban wastewater).

#### 2.5.4. Phosphate Adsorption Kinetic

The phosphate adsorption kinetic was evaluated using 25 mL of the synthetic phosphate solution, except for the SLMCT_2_ adsorbent which use a volume of 120 mL of solution at the same conditions. There were withdrawn samples (5 mL) at given times for controlling the phosphate concentrations and the pH in solution. The phosphate adsorption capacity as a function of time was calculated by Equation (2).

(2) $${\;Q}_{t}=\frac{\left(C_{o}-C_{t}\right)\times V}{w}$$

where Q_t_ is the equilibrium adsorption capacity (mg·g^−^^1^ PO_4_^3^^−^), V is the volume of solution (L), C_0_ and C_t_ are the initial and phosphate concentration at specific time (mg·L^−^^1^ PO_4_^3^^−^) and w is the mass of the adsorbent (g).

#### 2.5.5. Phosphate Fractioning

An adaptation of the three sequential-step phosphate extraction protocol was used [30]. Four fractions were quantified: labile, metal, alkaline and the residual phosphate. The phosphate adsorption was performed as described above in the previous assays. Once the supernatant was separated by centrifugation, the solid phase at the bottom of the centrifuge tube was collected, dried and stored for further tests. The labile phosphate fraction (loosely bound) was extracted from the solid phase (0.25 g) two successive times in 10 mL of 1 M NH_4_Cl (pH 7). The metal phosphate fraction (e.g., iron, manganese and aluminium) was obtained by two successive extractions in 10 mL of 0.1 M NaOH followed by extraction in 1 M NaCl. The phosphate alkaline fraction (e.g., sodium, magnesium and potassium) was extracted by two consecutive times in 10 mL of 0.5 M HCl. Finally, the remanent phosphate content was determined by mass balance between the phosphate adsorbed in adsorbents and the summatory of extracted fractions.

#### 2.5.6. Regeneration of Phosphate Saturated Adsorbents

The phosphate adsorption was performed as described above. Once the supernatant was separated by centrifugation, the solid phase at the bottom of the centrifuge tube was collected, dried and stored for further tests. The loaded adsorbents were equilibrated in aqueous solutions containing NaHCO_3_ (0.1 mol∙L^−^^1^ y pH 8.5). In the regenerated solutions it was determined the values of pH and phosphate concentration at initial and equilibrium state. The equilibrium desorption capacity was determined by Equation (3).

(3) $$Q_{d}=\frac{C_{e}\times V}{w}$$

where Q_d_ is the phosphate equilibrium desorption capacity (mg∙g^−^^1^ PO_4_^3^^−^), V is the volume of regeneration solution (L), C_e_ is the equilibrium phosphate concentration (mg∙L^−^^1^ PO_4_^3^^−^) and w is the mass of the adsorbent material used (g).

### 2.6. Phosphate Adsorption in Continuous Mode

The adsorbents (10 ± 0.2 g of PLMCT_3_ and SLMCT_2_) were packed in a glass column 3 cm diameter x 3 cm height. At the beginning the columns were equilibrated with ~20 BV of deionized water. The feed composition was established taking as reference the expected values of effluents streams of a wastewater treatment facility. The column was fed with a solution containing 10 mg∙L^−^^1^ PO_4_^3^^-^ at pH 7 ± 0.3 at a room temperature (21 ± 2 °C) at a feed rate of 1 mL∙min^−^^1^. There were withdrawn samples (5 mL) at given times for controlling the phosphate concentrations and the pH in solution. The solution was supplied in co-current through the column at EBHRT of 8 h.

## 3. Results

### 3.1. Physicochemical Propierties of Materials

The chemical composition of the materials used in this study are summary in Table 1. The presence of TiO_2_ and SnO_2_ were determined as minor components of MC and LMC adsorbents. The iron and manganese in LMC were tree times higher than MC.

The presence of cations (e.g., Mg^2+^, K^+^, Na^+^, Ca^2+^, data not shown) were verified by ICP in the exhausted loading solution (Table 2). Thus, ion exchange reaction occurred mainly by effect of Mg^2+^, Ca^2+^ followed by Na^+^ and K^+^ ions from MC that were exchanged with Fe^3+^ and Mn^2+^ from the loading solution. The K^+^ content in the exhausted loading solution was the lowest during the Fe-Mn loading stage because K^+^ from muscovite cannot be easily exchanged. The chemical composition of LMC, PLMCT_3_ and SLMCT_2_ composites were similar because any relevant change was determined.

**Figure 2:**
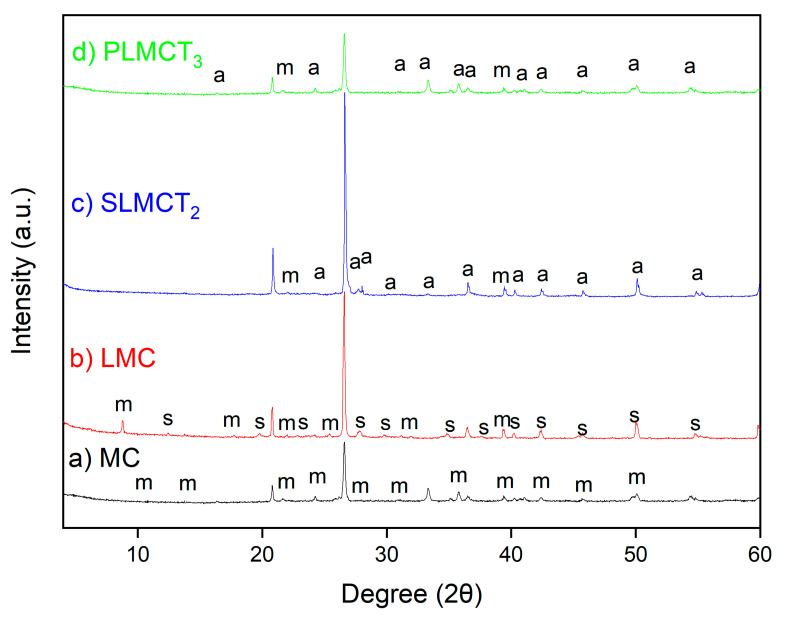
XRD patterns of the muscovite composites: (**a**) raw muscovite MC, (**b**) iron/manganese muscovite/sodalite LMC, (**c**) monolith muscovite/analcime SLMCT_2_ and (**d**) pellets muscovite/analcime composites PLMCT_3_. Nomenclature: m (muscovite), s (sodalite) and a (analcime).

The X-ray diffraction patterns of raw MC, LMC, PLMCT_3_ and SLMCT_2_ are depicted in Figure 2. The XRD patterns of materials are represented at wide angles (2θ: 4–60°). The MC in the raw form was a heterogeneous material composed by muscovite (M) [K_3.52_Na_0.44_Al_11.36_Fe_0.24_Mg_0.08_Si_12.32_O_48_H_8_] as the main mineralogical phase followed by quartz (Q) [SiO_2_]. The diffraction peaks of muscovite match well with the standard (Ref. Code 96-900-1957) at 2θ: 8.7° (002), 17.5° (004), 22.7° (11-3), 26.4° (024), 31.7° (11-6), 37.2° (027), 39.2° (117), 42.3° (04-3), 45.7° (029) and 50.1° (0210). The reflections of quartz (Ref. Code 96-900-9667) at 2θ = 26.47°, 42.11°, 54.50°, 59.47° and 67.74° [31]. The muscovite was indexed to the monoclinic crystal system and space group C 1 2/c1 with unit cell parameters a (Å): 5.22, b (Å): 9.05 and c (Å): 20.15. The basal space d_002_ plane was calculated as 10.07 Å for the raw MC at 2θ: 8.7, which was comparable to the d_002_ value of the muscovite pattern. The diffraction pattern of the LMC exhibited some changes in the position and intensity of the diffraction peaks of LMC in comparison to the raw MC. It was determined the formation of sodalite as new crystalline mineralogical phase following the muscovite, obtaining the muscovite/sodalite composite. Moreover, the simultaneous precipitation of iron–manganese hydroxide Fe(OH)_3_ (s) and Mn(OH)_2_ (s) nanoparticles by addition of NaOH (adjusting the pH 7.5) occurred over the surface of muscovite/sodalite composite; which was confirmed by SEM analysis. The partial dissolution of the Fe^3+^ and Mn^2+^ hydroxide nanoparticles M(OH) (s) into the ionic species M^+^ (aq) and OH^−^^1^ (aq) promote the coexistence of metal species in both forms M(OH) and M^+^.

The basal space (d_002_) of muscovite in the LMC form was 10.14 Å and an increase in the interlayer space (d_002_: 0.07 Å) were determined. The muscovite is a 2:1 layer phyllosilicate mineral composed by a crystal structure of two tetrahedral sheets—one dioctahedral sheet sandwiched between two tetrahedral sheets [32]. Hence, Si^4+^ and Al^3+^ of muscovite can be partially isomorphic replaced by low charge cations such as Fe^3+^ and Mn^2+^, which explain the slight changes in the DRX patterns of LMC. On other hand, the slight increase in the basal space suggested the partial incorporation of Fe^3+^ and Mn^2+^ in the lattices of muscovite promoting a small interlamellar expansion; but the interlamellar cation (e.g., K^+^) of muscovite cannot be easily exchanged. Finally, the incorporation of Fe^3+^ and Mn^2+^ can be also explained in terms of electrostatic attraction due to the negative charge of muscovite surface [15]. The diffraction peaks of sodalite Na_8_(Al_6_Si_6_O_24_)Cl_2_ match well with the standard (Ref. Code 96-900-5052) at 2θ: 13.8° (110), 19.8° (200), 27.8° (220), 34.8° (222), 37.5° (321), 40.1° (400), 42.3° (411), 45.7° (420), 50.0° (422) and 55.2° (521). The sodalite Na_8_Al_6_Si_6_O_24_Cl_2_ is a zeolite conventionally obtained by synthesis from silicon and aluminium sources (e.g., muscovite) [17]. The sodalite was indexed to the cubic phase and space group I a–3 d with unit cell parameters a (Å) = b (Å) = c (Å) = 9.009. The basal space d_110_ plane was calculated as 6.43 Å for the sodalite at 2θ: 13.8, which is comparable to the d_110_ value of the sodalite pattern 6.40 Å. The incorporation of Fe^3+^ and Mn^2+^ cations in the sodalite zeolite occurred in the extra-framework octahedral and in the framework tetrahedral sites as has been reported for other zeolites [14]. The information provided by the crystallographic parameters of the obtained sodalite suggest the partial incorporation of iron and manganese into the sodalite structure. Initially, Fe^3+^ and Mn^2+^ reached the extra-framework octahedral sites by outer complexation mechanisms (electrostatic attraction) with the negative charge over the surface of the sodalite. After, the addition of NaOH promoted the precipitation of Fe^3+^ and Mn^2+^ hydroxide nanoparticles and their further dissolution into Fe^3+^ and Mn^2+^ allowing their incorporation to the tetrahedral framework sites via isomorphic substitution [17]. The increase in the intensity and the well-defined peaks of LMC in comparison to the raw MC can be explained in terms of the crystallinity of the new muscovite/sodalite composite structure. However, the higher number of extra-framework sites of sodalite due to the incorporation of Fe^3+^ and Mn^2+^ in the cages do not affect their structure [33].

The diffraction patterns of the SLMCT_2_ and PLMCT_3_ exhibited new changes in the position and intensity of the diffraction peaks in comparison to the muscovite/sodalite composite LMC. The existence of quartz and muscovite were corroborated in SLMCT_2_ and PLMCT_3_. However, it was determined the analcime as major and recently formed crystalline mineralogical phase, of the obtained muscovite/analcime composite (monoliths and pellets). The diffraction peaks of analcime zeolite type (NaAlSi_2_O_6_H_2_O) match well with the standard (Ref. Code 96-900-4014) at 2θ: 16.3° (211), 25.9° (400), 30.9° (332), 33.3° (431), 36.5° (440), 40.2° (611), 40.9° (620), 42.3° (541), 45.7° (444), 49.7° (642) and 54.3° (741). The analcime was indexed to the cubic phase and space group I a–3 d with unit cell parameters a (Å) = b (Å) = c (Å) = 13.8. The basal space d_211_ plane was calculated as 5.42 Å for the analcime at 2θ: 16.3, comparable to the d_211_ value of the analcime pattern 5.39 Å. The obtaining of analcime has been reported to occur in several condition of synthesis (e.g., different silicon and aluminium sources, Si/Al ratios, temperature and pressure ranges). However, most of the sources used for synthesis do not provide high purity of analcime crystalline phase; thus, the product can contain additional zeolitic phases or fractions of raw materials as occurred in this study. Several zeolites are known to maintain their crystal framework at elevated temperatures such as sodalite, analcime or faujasite. However, information about the influence of high-temperatures on the behaviour of zeolites has not been easily found. Nevertheless, thermally induced dehydroxylation promotes several transformation types, such as amorphization, recrystallization and dealumination [34]. Thus, the occurrence of successive phase transformation of zeolites may explain the formation of more stable phases such as analcime, promoted by higher amounts of silicon in dissolution [35] during synthesis at higher temperatures as occurred in this study. The formation of the analcime zeolite depends on various factors such as the composition of the parent material, crystallisation temperature, cation concentrations and pH. However, information about the obtainment of analcime zeolite from sodalite phase after calcination has not been easily found. The muscovite/sodalite composite as parent material used in this study, due to its chemical composition (e.g., K, Mg, Ca, Na) and the pH of the alkaline fluid phase (e.g., pH 7) at the activation temperature, promoted the optimal conditions for the obtainment of the muscovite/analcime composite. The alteration of the basaltic glasses of the muscovite/sodalite composite structure during the thermal activation allowed the transformation into the muscovite/analcime phase of the monoliths and pellets [36]. The information provided by the crystallographic parameters of the obtained analcime also suggests that Fe^3+^ and Mn^2+^ are partially incorporated into the analcime structure following the occupancy of the extra-framework octahedral and the framework tetrahedral sites; mechanisms that were above-discussed for sodalite. The diffractogram spectra of the muscovite/analcime composites PMLCT_3_ and SLMCT_2_ differed in their intensity and crystallinity. The starting materials, the preparation of the composites, the Fe^3+^/Mn^2+^ incorporation into the structure and the temperature determined the crystalline symmetry of the obtained materials [37,38].

The surface area value of raw MC was 7.0 m^2^ g^−^^1^, comparable with the reported value for other muscovite materials [17]. The surface area of the muscovite/sodalite powder composite LMC increased to 74.0 m^2^.g^−^^1^, the sodalite as zeolitic phase and the incorporation of Fe^3+^/Mn^2+^ (oxy)hydroxide nanoparticles onto the muscovite/sodalite composite is associated to a larger availability of bonding sites. The Fe^3+^/Mn^2+^ incorporated to muscovite by isomorphic replacement, cation electrostatic, precipitation and complexation reactions produce a higher surface area. The obtaining of high crystalline sodalite zeolite by itself has a high surface area, and the Fe^3+^/Mn^2+^ incorporation at the extra-framework octahedral followed by the occupation of the framework tetrahedral sites improved this property. However, a sharp reduction in surface area was experimented for the monoliths and pellets in comparison to the powder LMC. There were determined specific surface area values of 2 m^2^.g^−^^1^ and 1 m^2^.g^−^^1^ for PLMCT_3_ and SLMCT_2_, respectively. The thermal treatment promoted the reduction in surface area due to the dihydroxylation, characterized by the elimination of physical adsorbed water and the hydroxyl groups of the aluminosilicate surface (e.g., muscovite, sodalite); it will be corroborated by FTIR analysis. The analcime zeolite of PLMCT_3_ and SLMCT_2_ was characterized by a close-pack structure with a small pore diameter that makes the diffusion of molecules (e.g., nitrogen) difficult, developing lower area than sodalite zeolite found in LMC [39]. The surface area values reported for composite monoliths (SLMCT_2_) and pellets (PLMCT_3_) are comparable to those reported for a synthesized analcime with high crystallinity and low porosity [37].

**Figure 3:**
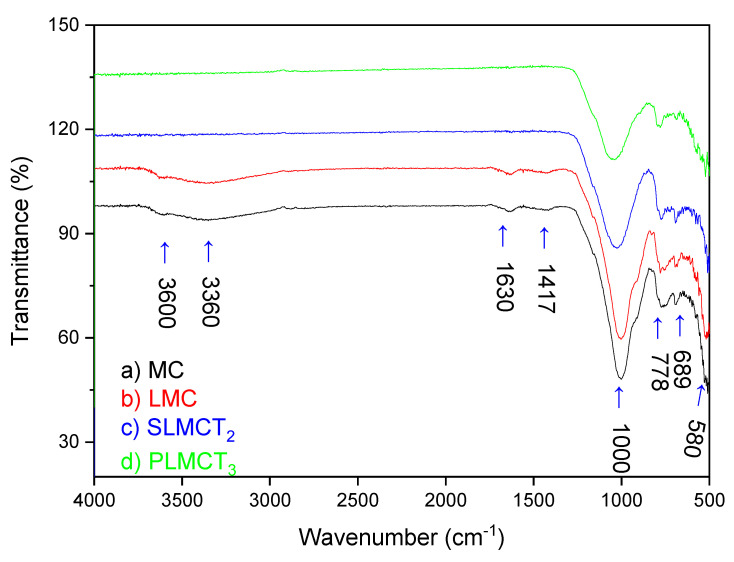
FTIR spectra of the adsorbents: (**a**) raw muscovite MC, (**b**) iron/manganese muscovite/sodalite LMC, (**c**) monolith muscovite/analcime SLMCT_2_ and (**d**) pellets muscovite/analcime composites PLMCT_3_.

The FTIR spectra of the materials used in this study are represented in Figure 3. The characteristic absorption bands of muscovite were clearly identified. The absorption band at 3600 cm^−^^1^ was attributed to the internal –OH groups (physical adsorbed water molecules); while the absorption band at 3360 cm^−^^1^ was attributed to the H–O–H stretching adsorbed water of muscovite [40]. The band at 1630 cm^−^^1^ was the indicative for the H–O–H bending vibration of adsorbed water on the muscovite; while the band at 1417 cm^−^^1^ was associated as the characteristic peak for the H–O–H bending of water on the raw MC [39]. The existence of the absorption band at 1000 cm^−^^1^ in the raw MC represented the stretching vibrations of Si–O and Al–O tetrahedra. The peak at 778 cm^−^^1^ in the raw MC confirmed the bending vibration of Al–O–H and the Si–O–Al bond at 689 cm^−1^ [17]. The existence of the absorption band at 580 cm^−^^1^ confirmed the vibration of Si–O–Si of MC [41]. The FTIR spectra of the muscovite/sodalite composite LMC has the same absorption bands of MC with some changes in the intensity and positions at 3630, 3320, 1625, 1431, 1005 and 790 cm^−^^1^. Important changes were determined at four different regions characteristic of the sodalite zeolite. The shift of low bands at 513, 542 and 562 cm^−^^1^ was identified, the asymmetric stretch of Fe–O and the bending vibrations of Al–OH bonds are involved; attributed to the isomorphic substitution of Fe^3+^, Mn^2+^ into the sodalite structure. Changes were also evidenced at absorption bands in the region between 500 and 700 cm^−^^1^ corresponding to the symmetric T–O–T (where T: Si or Al) stretching vibrations (*ν_s_*). The displacement of absorption bands of the asymmetric T–O–T stretching vibrations (*ν_as_*) occurred between 701 and 790 cm^−^^1^. The shift of bands at 697 and 778 cm^−^^1^ revealed changes in the muscovite structure due to the transformation into sodalite. The shift of band at 1014 cm^−^^1^ belongs to the stretching modes of Si–O–Si. The appearance of new bands at 607, 683 and 757 cm^−^^1^ are related to siloxane groups (Si–O–Al and Si–O–Si bonds), attributed to the connection of SiO_4_ or AlO_4_ tetrahedron vibration, associated with the metakaolinization process during the zeolite synthesis to sodalite [39]. The shift of bending (at 1625 and 1637 cm^−^^1^) and stretching vibration of water (at 3320 and 3630 cm^−^^1^); were attributed to the stabilizing effect of water in the sodalite cages [42]. The shift of the absorption bands of –OH groups were also attributed to the incorporation of Fe^3+^/Mn^2+^ (oxy)hydroxide nanoparticles onto the LMC by inner sphere complexation reactions [39] The incorporation of Fe^3+^/Mn^2^ at the extra-framework octahedral (outer sphere complexation) and the framework tetrahedral (inner sphere complexation) sites also promoted some structural changes in sodalite [17]. Though, it has not been identified specific absorption bands that revealed the existence of exchange ions (e.g., Fe^3+^, Mn^2+^) in the sodalite framework. However, the changes found in the FTIR spectrum of muscovite/sodalite composite between the absorption bands 830 and 880 cm^−^^1^; could be associated with the existence of some metal ions as occurred on a sodalite theoretical studied [43].

The FTIR spectra of muscovite/analcime composites PLMCT_3_ and SLMCT_2_ revealed main absorption bands at 3605, 3327, 1635, 1421, 1009, 777, 743, 693, 606 and 524 cm^−^^1^. The FTIR spectra of muscovite/sodalite composites (PLMCT_3_ and SLMCT_2_ spectra) revealed the shift and appearance of new bands. The shift of low absorption bands of SLMCT_2_ (at 524, 534, 545, 560 and 573 cm^−^^1^) and for PLMCT_3_ (at 523, 549 and 569 cm^−^^1^) were attributed to the partial isomorphic substitution of Fe^3+^, Mn^2+^ into the analcime’s structure due to the asymmetric stretch of Fe-O and the bending vibrations of Al-OH bonds. The shift of bands of SLMCT_2_ (at 744, 773, 795 and 799 cm^−^^1^) and PLMCT_3_ (at 734, 747, 781 and 793 cm^−^^1^) are attributed to the asymmetric T-O-T stretching vibrations. The shift of the band at 998 cm^−^^1^ for SLMCT_2_ and 1110 cm^−^^1^ for PLMCT_3_ belong to the stretching modes of Si-O-Si [39]. Thus, SiO_4_ or AlO_4_ tetrahedron vibration by the T-O-T groups arrangement occurred during the synthesis of analcime zeolites as it has been previously reported. The most important difference in the FTIR spectra between muscovite/analcime composite and muscovite/sodalite composite occurred in the absorption bands of molecular water (3300 and 3600 cm^−^^1^). The shift of bands of SLMCT_2_ (1653, 3630 and 3339 cm^−^^1^) and PLMCT_3_ (1637 and both 3620 and 3381 cm^−^^1^; that almost disappear) have been associated with the transformation of a zeolitic phase into another, because the water absorption bands disappear gradually with the increase in temperature. The release of the zeolite water occurred during the transformation of zeolite phase without promoting relevant changes in the crystal structure [44]; as occurred in this study. In addition, the absence of OH absorption bands suggested the existence of porous zeolite cage structures without occluded water molecules [45]. The changes discussed above were also promoted by the incorporation of Fe^3+^/Mn^2+^ (oxy)hydroxide onto the SLMCT_2_ and PLMCT_3_ composites by inner sphere complexation reactions. In conclusion, the FTIR spectra of LMC, PLMCT_3_ and SLMCT_2_ specifically revealed the modification of the absorption bands related to the formation of new zeolitic phases in the muscovite composites prepared in this study and the existence of (≅FeOH) and (≅MnOH) groups as functional sites for further phosphate adsorption in a greater or lesser extent.

**Figure 4:**
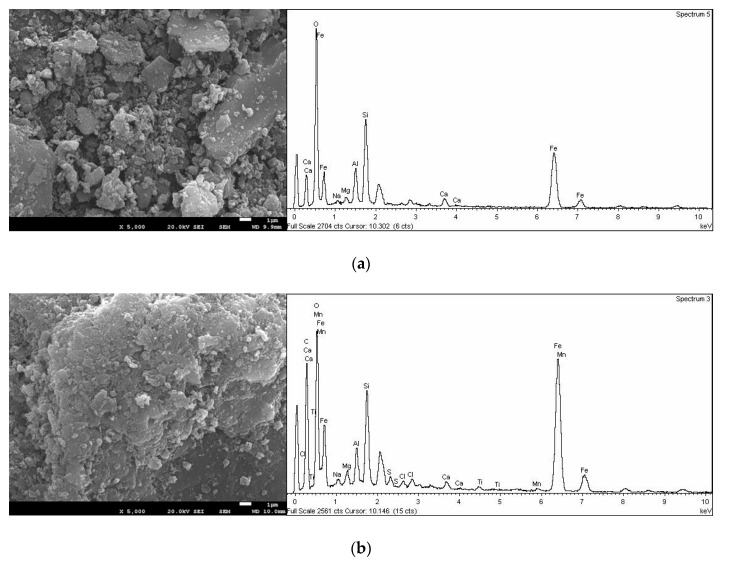

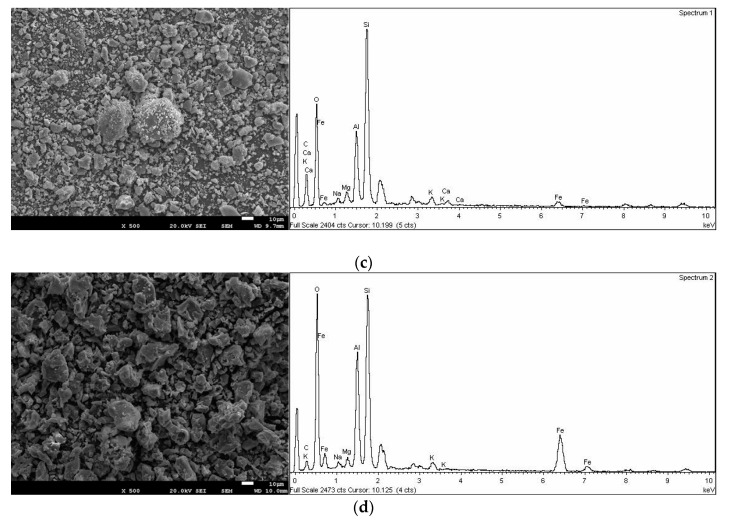
FSEM—EDX of the adsorbents: (**a**) raw muscovite MC, (**b**) iron/manganese muscovite/sodalite LMC, (**c**) monolith muscovite/analcime SLMCT_2_ and (**d**) pellets muscovite/analcime composites PLMCT_3_.

The FSEM—EDX of the adsorbents used in this study are displayed in Figure 4. The raw muscovite MC surface appeared as rough heterogeneous grains crystalline morphology. The muscovite grains seem to be obtained by fragmentation of a larger plate at regular intervals [40]. The crystal size of the muscovite plates based on SEM were estimated to be in the range of 0.1 to 7 μm.

The colour of MC turned yellow after being obtained the Fe^3+^/Mn^2+^ muscovite/sodalite composite (LMC). Moreover, FSEM-EDX (Figure 4b) revealed a layer of precipitates covering the surface of MC, attributed to the new zeolitic phase synthesized and the incorporation of iron and manganese (oxy)hydroxides nanoparticles over MC. The surface of LMC exhibited a new morphology, rougher than the raw MC surface. The morphology of LMC also exhibited the sodalite crystals appeared as octahedral grains forming flower-like shapes clusters precipitated over the raw muscovite MC similar to those reported in the literature. The crystal size of the sodalite based on SEM was estimated to be in the range of 0.3 to 2 μm [17,34]. The SLMCT_2_ and PLMCT_3_ morphology demonstrate the analcime formation as a new mineralogical phase with poorly defined crystalline faces as it has been reported before. In both cases SLMCT_2_ and PLMCT_3_ coexist with the muscovite and silica aggregates of the raw material MC [46]. Over the surface of LMC, SLMCT_2_ and PLMCT_3_ there were determined the existence of small particles, which are attributed to the thin layer of iron—manganese (oxy)hydroxide as functional groups further phosphate adsorption.

### 3.2. Influence of the Calcination Temperature on the Phosphate Adsorption

**Figure 5:**
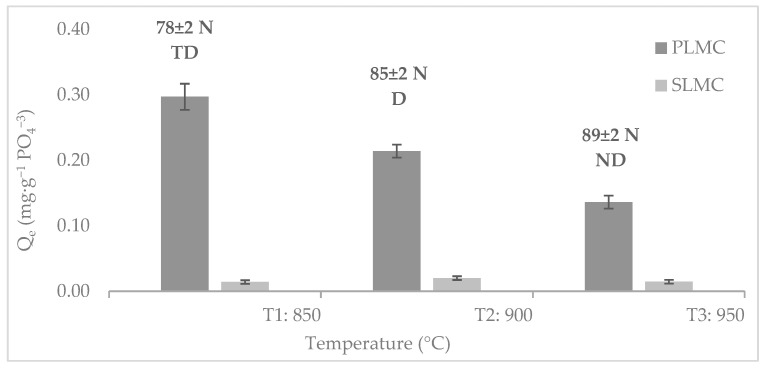
Phosphate adsorption capacity and resistance force of adsorbents as a function of calcination temperature of composites. Parameters obtained at V: 25 mL, w: 0.25 g and C_i_: 10–2000 mg·L^−^^1^ PO_4_^3^^−^; except for SLMC, w: 10 ± 0.2 g was used.

The effect of the calcination temperature on the phosphate adsorption and the resistance force of adsorbents are depicted in the Figure 5. The phosphate adsorption capacity of PLMC was 20 times higher than SLMC at overall temperatures even low masses of PLMC were used at overall assays. PLMC is totally composed by loaded Fe^3+^/Mn^2+^ muscovite/zeolite composite, while SLMC was prepared by impregnation on a polymeric scaffold. The mass of loaded Fe^3+^/Mn^2+^ muscovite/zeolite composite per gram of adsorbent was higher in the PLMC than in SLMC composite. Decreases in phosphate adsorption of 28 and 32% occurred with the increase in temperature for PLMC composite to 900 and 950 °C, respectively. The phosphate adsorption capacity onto the SLMC composite remained invariable along the temperature. In this stage, the lower phosphate adsorption capacity of SLMC (1 m^2^.g^−^^1^ ) in comparison to PLMC (2 m^2^.g^−^^1^) can be attributed to the surface area as one of the physicochemical property. Particularly, the reduction in surface area of SLMC was promoted by the effect of pore blockage due to increase in the material thickness around the polymeric scaffold at the sintering temperatures [47]. Thus, in SLMC composite only the active phase of the loaded Fe^3+^/Mn^2+^ muscovite/zeolite composite takes part of the phosphate adsorption being the rest inert.

On the other hand, the resistance of composite monoliths (SLMC) increased with the temperature; however, the highest phosphate adsorption was determined for the sample prepared at T_2_: 900 °C; which is the optimal temperature for the preparation of composite monoliths. The composite pellets (PLMC) calcined at 950 °C did not disaggregate in the aqueous phosphate solution in comparison to those obtained at lower temperatures 850 °C and 900 °C. The high temperature increased the hydrophobic nature of adsorbents. It was established T_3_: 950 °C as optimal temperature for composite pellets preparation, even though the lowest phosphate adsorption capacity was obtained. Thus, the pellets PLMCT_3_ and monoliths SLMCT_2_ were used for phosphate adsorption due to their stability and resistance force necessary for further essays in batch and fixed-bed disposal. A high resistance force is desirable for adsorbents packing achieved at high temperatures without the surface become glassy. Conventionally, the high resistance force is promoted by the densification of ceramic foam by stronger bonding of ceramic components [47]. However, the methods of preparation determined the mechanical strength of the obtained form of densified materials [27].

### 3.3. Effect of the pH on Phosphate Removal

**Figure 6:**
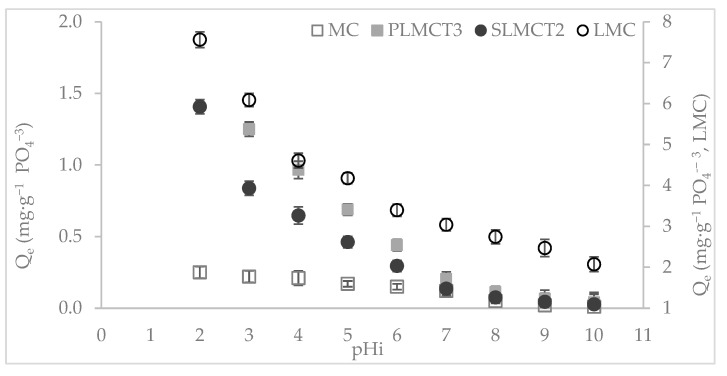
Phosphate adsorption capacity of adsorbents as a function of pH of solution. Values obtained at V: 25 mL, w: 0.25 g and C_i_: 10–2000 mg·L^−^^1^ PO_4_^3^^−^; except for SLMCT_2_ w: 10 ± 0.2 g.

The phosphate adsorption is dependent of the pH of the solution as depicted in Figure 6. The phosphate adsorption capacity of raw muscovite MC was improved with the obtaining of muscovite/sodalite composites and the incorporation of Fe-Mn (oxy)hydroxide nanoparticles (LMC). The highest adsorption capacity values were provided by LMC under the overall pH essays. The phosphate adsorption capacity of the muscovite/analcime pellets (PLMCT_3_) were higher than the muscovite/analcime monoliths (SLMCT_2_); even though low amount of adsorbent PLMCT_3_ were required. The phosphate adsorption capacity onto the adsorbents (MC, LMC, PLMCT_3_ and SLMCT_2_) is fully dependent of the pH of the solution and they followed similar trend. The values of the point of zero charge of the adsorbents were determined to be pH_PZC_: 6.8 ± 0.1, 7.8 ± 0.1, 7.4 ± 0.1 and 7.5 ± 0.1 for MC, LMC, PLMCT_3_ and SLMCT_2_, respectively. The values of the point of zero charge of this study were comparable with those reported for other adsorbents in their raw and modified state [48]. A slight increase in the value of the point of zero charge of muscovite/sodalite composite LMC occurred in comparison to the raw muscovite MC. The change in the pH_PZC_ was attributed to the obtaining of new physicochemical properties in the adsorbents. The obtaining of muscovite/zeolite composites and the incorporation of Fe-Mn (oxy)hydroxide nanoparticles also favoured the phosphate adsorption capacity. The phosphate adsorption capacity onto Fe^3+^/Mn^2+^ (oxy)hydroxide nanoparticles muscovite/sodalite composite (LMC) increased twenty-fold over MC at pH 7. At the same conditions, the adsorption capacity of PLMCT_3_ and SLMCT_2_ were almost the same in comparison to the raw muscovite (MC). The highest phosphate adsorption capacity values were obtained at acid pH zone between pH 2 and 7 (below pH_PZC_) and the reduction in the adsorption capacity values occurred in the range between pH 8 and 10 (above pH_PZC_).

Below the pH_PZC_, the H_2_PO_4_^−^ and HPO_4_^2^^−^ anionic forms of phosphate interacted with the positive electric field, promoted by the protonation of iron hydroxyl groups. It is explained in terms of the high basicity of phosphate anions (HPO_4_^2^^−^) with high electronic density they formed hydrogen bonds with the protonated Fe–(OH)^+^ and Mn–(OH)^+^ groups of the adsorbents [11,49]. On the other hand, the hydroxylation of the Fe–(OH)^+^ and Mn–(OH)^+^ groups occurred above the pH_PZC_. Then, the competition of the phosphate oxyanions specie (e.g., HPO_4_^2^^−^) and the hard Lewis base (OH^−^ ions) occurred at the surface of the adsorbents [50], promoting the reduction in the adsorption capacity. The occurrence of these electric interaction forces are denoted as physisorption or outer-sphere adsorption complexes [10]. In comparison to other phosphate adsorbents, the advantages of the adsorbents (MC, LMC, PLMCT_3_ and SLMCT_2_) allow phosphate removal at the usual pH condition of treated wastewater (e.g., pH 7). Therefore, the phosphate recovery using the adsorbents (MC, LMC, PLMCT_3_ and SLMCT_2_) from wastewater treatment plants could be performed without pH adjustment requirements [13].

### 3.4. Isotherms of Phosphate Adsorption onto the Adsorbents

A broad range of phosphate concentrations were evaluated for adsorption to demonstrate the sensitivity of the adsorbents (MC, LMC, PLMCT_3_ and SLMCT_2_). An easier mass transfer of phosphate occurred from aqueous phase to solid material surface since higher phosphate concentration provided higher driving forces [11]. There were determined maximum adsorption capacities as the most important physicochemical parameters to evaluate the performance of adsorbents [51]. The phosphate adsorption of muscovite/sodalite composite LMC was three times higher than raw MC. The phosphate adsorption capacity of LMC increased seven- and thirty-fold over PLMCT_3_ and SLMCT_2_ composites, respectively. The phosphate adsorption capacity of MC was two times higher than the PLMCT_3_ and SLMCT_2_ composites. The efficiency of phosphate adsorption onto powder adsorbents MC and LMC were higher than the densified adsorbents PLMCT_3_ and SLMCT_2_. The effect of densification of powders and the temperature promoted the change in physicochemical properties (mainly surface area) modifying their starting properties and their phosphate adsorption capacities. However, the PLMCT_3_ and SLMCT_2_ become prominent materials for operation in fixed-bed column, in comparison to the MC and LMC materials which are viable materials for stirred-tank applications.

The experimental data of phosphate adsorption were adjusted to the Langmuir and Freundlich isotherms (Table 3). The linearised Langmuir isotherm equation (Equation (4)) considered $Q_{m}$ as the maximum adsorption capacity (mg⋅g^−^^1^ PO_4_^3^^−^), $K_{L}$ Langmuir adsorption constant (L⋅mg^−^^1^). The linearised Freundlich isotherm equation (Equation (5)) considers $K_{F}$ (mg⋅g^−^^1^) and n as Freundlich constants.

(4) $$\frac{C_{e}}{Q_{e}}=\frac{C_{e}}{Q_{m}}+\frac{1}{K_{L}Q_{m}}$$

(5) $$\ln Q_{e}={\ln\;K}_{F}+\frac{1}{n}{\mathrm{lnC}}_{e}$$

The data were best fitted to the Langmuir isotherm model, R^2^ ≈ 1, revealing the occurrence of monolayer adsorption. Phosphate is adsorbed on specific equivalent and identical bonding sites [52]. The experimental data of phosphate adsorption onto adsorbents used in this study were not well fitted to the Freundlich isotherm model with values of 0.74 ≤ R^2^ ≤ 0.90. The heterogenous surface of the adsorbents used in this study, conventionally are associated with heterogenous surface energy active according to the Freundlich isotherm model [53]. Thus, the phosphate adsorption onto the adsorbents (MC, LMC, PLMCT_3_ and SLMCT_2_) was mainly governed by specific adsorption, followed by non-specific adsorption, as was discussed in Section 3.3.

**Figure 7:**
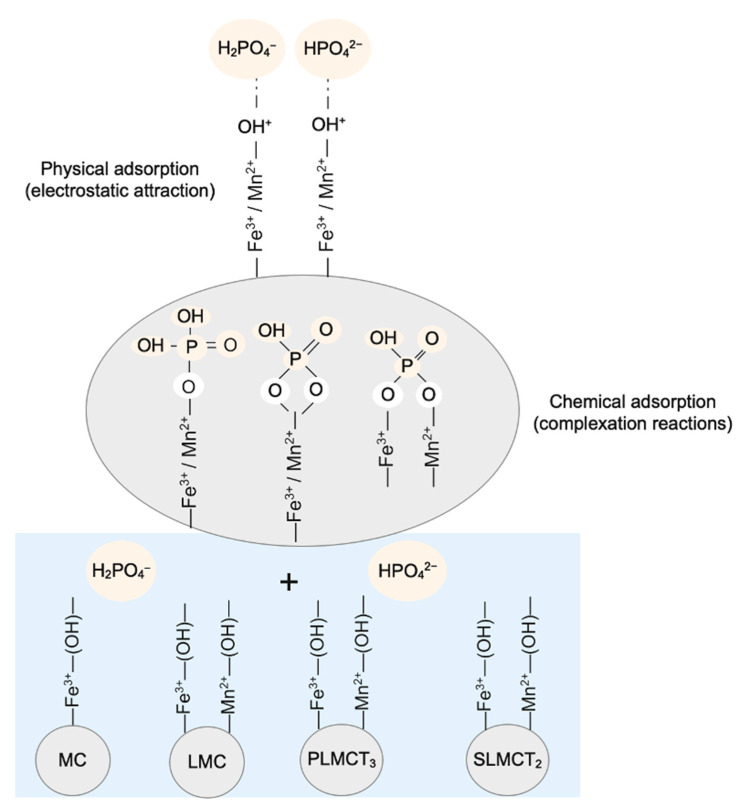
Governing mechanisms of phosphate adsorption onto the loaded Fe^3+^/Mn^2+^ muscovite/zeolite composites (powder, pellets and monoliths). Schematical representation of the phosphate adsorption.

The isotherm parameters suggest that specific phosphate adsorption onto adsorbents (MC, LMC, PLMCT_3_ and SLMCT_2_) could be attributed to the Fe–Mn surface hydroxyl groups. The raw muscovite is composed by hydroxyl groups (e.g., Fe, Al); but the higher content of Fe^3+^ and Mn^2+^ hydroxyl groups on LMC, promoted the improvement of phosphate adsorption. The phosphate adsorption onto adsorbents can be explained in terms of the protonation of Fe–(OH)^+^ and Mn–(OH)^+^ groups which can be replaced by the phosphate anionic species. The inner sphere complexation reactions promoted the formation of monodentate or bidentate forms. The occurrence of physical adsorption (outer sphere) and chemical adsorption (inner sphere) reactions explained the phosphate adsorption. The mechanisms described above are schematically represented by Figure 7 [9].

**Figure 8:**
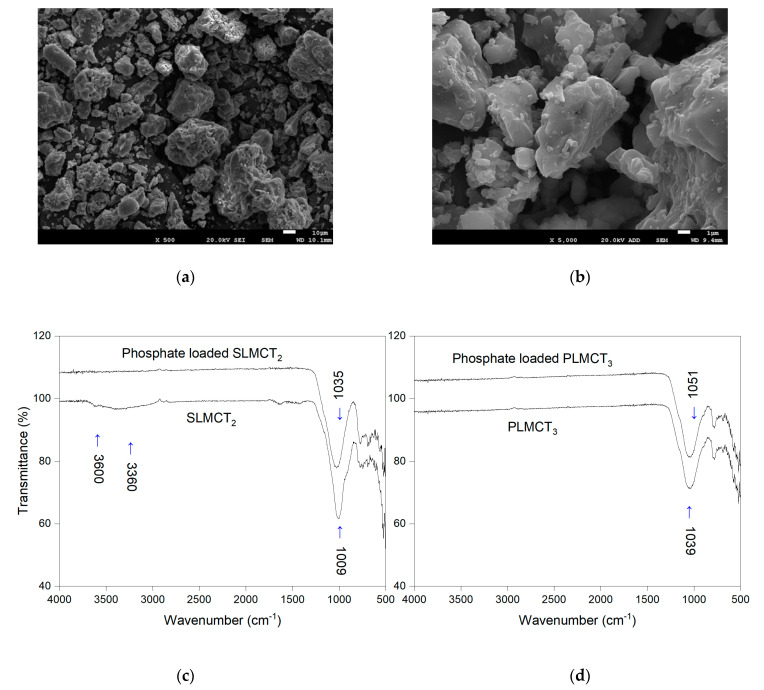
SEM photography of the adsorbents after phosphate adsorption: (**a**) pellets muscovite/analcime composites PLMCT_3_, (**b**) monolith muscovite/analcime SLMCT_2_ and comparison of FTIR spectra between composites before and after phosphate adsorption: (**c**) monolith muscovite/analcime (SLMCT_2_) and (**d**) pellets muscovite/analcime composites (PLMCT_3_).

The proposed mechanisms for phosphate adsorption were verified by means of both SEM and FTIR characterization techniques (Figure 8). The morphology of the saturated loaded Fe^3+^/Mn^2+^ muscovite/zeolite composites demonstrate the existence of particles deposited over the composite surface (Figure 8a,b). The increase in the roughness over the zeolites surfaces after phosphate adsorption also was determined. On the other hand, the FTIR spectra of the saturated composites (Figure 8c) revealed phosphate adsorption on the loaded Fe^3+^/Mn^2+^ muscovite/zeolite composites (PLMCT_3_ and SLMCT_2_). The shift at the absorption bands (1035 and 1051 cm^−^^1^ for SLMCT_2_ and PLMCT_3_, respectively) are characteristic of the Si–O–Si groups [54], revealing phosphate adsorption. The disappear of the broad band between 3400 and 3600 cm^−^^1^ was characteristic of phosphate adsorption in the Fe–(OH)^+^ and Mn–(OH)^+^ groups. Thus, the protonation of metal—(OH)^+^ group promote phosphate adsorption by outer sphere and inner sphere reactions according to the discussed mechanisms.

### 3.5. Kinetic of Phosphate Adsorption onto Adsorbents

**Figure 9:**
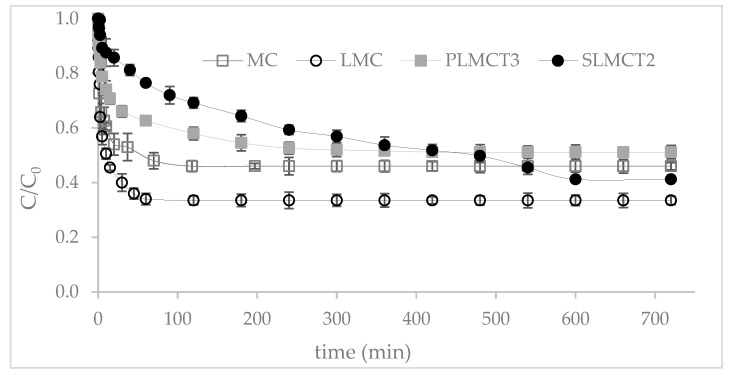
Phosphate adsorption kinetic of the adsorbents. Values obtained at V: 25 mL, w: 0.25 g and C_i_: 25 mg·L^−^^1^ PO_4_^3^^−^; except for SLMCT_2_ w: 10 ± 0.2 g and V: 120 mL.

The kinetic profile of phosphate adsorption is depicted in Figure 9. The equilibrium of phosphate adsorption was reached within 30 min for the powder MC and LMC. Higher removal rate (66%) was reached by LMC in comparison to 54% of MC. Larger time intervals were necessary for the muscovite/analcime composites (PLMCT_3_ and SLMCT_2_) to reach the equilibrium. The equilibrium attainment of phosphate adsorption was reached within 150 min with a phosphate removal rate of 46% for PLMCT_3_ and 59% of removal for SLMCT_2_. Higher mass of adsorbent and volume of phosphate solution were used for phosphate removal on SLMCT_2_. The slow phosphate adsorption can be explained in terms of difficult access to the binding sites Fe–(OH)^+^ and Mn–(OH)^+^ of the densified adsorbents; as well as the low content of Fe–(OH)^+^ and Mn–(OH)^+^, as demonstrated by the FTIR analysis. In other words, the low performance of the adsorbents is associated with the low and difficult access to the specific bonding sites of adsorbents. The effectiveness of phosphate removal is not always conditioned by the surface area of an adsorbent material; for example, the kinetic performance of MC and LMC composites are comparable with other mesoporous materials with higher surface area [55]. Therefore, phosphate adsorption is not only conditioned by surface mechanisms.

The experimental data of phosphate adsorption on adsorbents were adjusted to the pseudo-first and pseudo-second order kinetic models Table S1 [56]. Physisorption and chemisorption were established as main adsorption mechanisms. The pseudo-first and second order kinetic modelling revealed a R^2^ ≈ 1. The intraparticular diffusion kinetic model also described well (R^2^ closer to 1) phosphate adsorption onto the adsorbents. Phosphate adsorption from aqueous solution to a solid-phase interface is well explained in terms of adsorbate diffusion-controlled in macroscopic adsorbent particles. The experimental data exhibited a multi-linear plot; thus, more than two steps influenced phosphate adsorption process.

The experimental data were also fitted to the Shell Progressive Model (SPM) and the Homogeneous Diffusion Model (HDM) and summary in Table 4. The SPM model established the porosity of the adsorbents were small and practically impervious to the aqueous solution. Then, the adsorption process could be described by a concentration profile of the phosphate anions going forward into a spherical partially saturated particle [57]. The fluid film [*K_F_* (m·s^−^^1^)] is the adsorption rate-controlling step on the adsorbents particle, defined by linear Equation (6). The diffusion through the particle adsorption layer [*D_p_* (m^2^·s^−^^1^)] controlling the adsorption rate is described by the linear Equation (7). Finally, the chemical reaction [*k_s_* (m·mol·L^−^^1^·s^−^^1^))] controlling the adsorption rate is described by the linear Equation (8). The *X(t)* denotes the fractional attainment of adsorption equilibrium between the solid and liquid phase (Q_t_/Q_e_) at time *t*, *t* is the contact time (min) and *C_c_* and *C_s0_* (mg·L^−^^1^) are the concentration of solute at adsorbents unreacted core and in bulk solution, respectively; and a_s_ is the stoichiometric coefficient.

(6) $$X\left(t\right)=\frac{3C_{s0}K_{F}}{a_{s}rC_{c}}\;t$$

(7) $$\left[3-3{\left(1-X\left(t\right)\right)}^{2/3}-2X\left(t\right)\right]=\frac{6D_{p}C_{s0}}{a_{s}r^{2}C_{c}}\;t$$

(8) $$\left[1-{\left(1-X\left(t\right)\right)}^{1/3}\right]=\frac{k_{s}C_{s0}}{r}\;t$$

The adsorbents are considered as a quasi-homogeneous media is defined by the HDM model by the adsorption diffusion rate as controlling step on the spherical particles. The adsorption rate controlled by particle diffusion *D_p_* (m^2^·s^−^^1^) is defined by linear Equation (9). The liquid film diffusion *D_f_* (m^2^·s^−^^1^) controlling the adsorption rate is described by linear Equation (10) [57]. The h is the thickness of film around the adsorbents particle (1 × 10^−^^5^ m for a poorly stirred solution) and r is the average radius of adsorbents particles (particles below 200 mesh ≈ particles diameter: 7.4 × 10^−^^5^ m or particles radius: 3.7 × 10^−^^5^ m), and *C* and *C_r_* (mg·L^−^^1^) are the concentrations of solute in a solution and the adsorbent phase, respectively [58].

(9) $$-\ln\left(1-X{\left(t\right)}^{2}\right)=k_{p}t=\frac{2\;\pi^{2}D_{p}}{r^{2}}t$$

(10) $$-\ln\left(1-X\left(t\right)\right)=k_{f}t=\frac{D_{f}C}{h\;r\;C_{r}}t$$

The R^2^ values of the linear regression of the adsorption rate equation of the Homogeneous Diffusion Model (HDM) and Shell Progressive Model (SPM) were closer to 1. The effective diffusion coefficients (*D_p_* and *D_f_*) reached values in the order of 10^−^^15^ to 10^−^^7^ m^2^.s^−^^1^. The obtained values were comparable with the obtained for clays and zeolites adsorbents [59]. The kinetic performance of adsorbents (e.g., slow or fast) is determined by the phosphate adsorption mechanisms that governed the system [60]. Phosphate adsorption rate on the adsorbents were controlled by specific and consecutive phases. At the beginning a fast phosphate adsorption rate occurred on the surface of the adsorbents till the saturation. Phosphate anion diffused through the internal pores of the adsorbents with a slower adsorption rate. The occurrence of electrostatic attraction reactions (physical adsorption) were attributed to the fast phosphate adsorption rate stage. The second stage was attributed to phosphate complexation reaction since chemical adsorption occurred slow with high energy requirements.

The kinetical parameters determined for powder raw muscovite MC and muscovite/sodalite composites LMC suggest the application in stirred reactor-based arrangements. Even though, higher phosphate removal efficiencies have been reported for powder clays and zeolites [5,14]. The fixed-bed column adsorption arrangement is conventionally limited for powders, but viable for muscovite/analcime composites PLMCT_3_ and SLMCT_2_. Though, high efficiencies for phosphate removal have been reported by polymeric exchangers at low levels [13]. In this case, the use of PLMCT_3_ and SLMCT_2_ can be focused on the treatment of short volumes of urban wastewater with low concentration of phosphate [13]. The convenience of the adsorbents used in this study (MC, LMC, PLMCT_3_ and SLMCT_2_) for soil amendment applications and the final disposal recommendation is further corroborated by the fractioning and the regeneration essays.

### 3.6. Phosphate Fractioning from Doped Adsorbents

The fraction of phosphate bonded to the adsorbents are summary in Table 5. The labile fraction of phosphate (LB–P) was around 30–35%. The loosely bonded fraction represents phosphate immobilized by means of physical adsorption (electrostatic interactions), and is the portion of phosphate that can be available for plants. The second fractions bonded to metallic species (e.g., Al^3+^) Fe–(OH)^+^ and Mn–(OH)^+^ hydroxide are between 39 and 48%. This fact corroborates the chemical adsorption of phosphate to the metal (oxy)hydroxide (e.g., Fe–(OH)^+^ and Mn–(OH)^+^) sites of the MC, LMC, PLMCT_3_ and SLMCT_2_. The inner sphere complexation, as a chemical mechanism and conventionally irreversible, is hard to extract. The alkaline fractions of phosphate bonded to adsorbents are between 6 and 9%. Phosphate fraction immobilized by precipitation reactions are conventionally bonded to cations (e.g., Mg^2+^, K^+^, Na^+^, Ca^2+^). However, any new mineralogical phase was detected in the DRX analysis of the adsorbents. Finally, the residual fractions of phosphate bonded to the adsorbents were around 10–22%.

No comparable information about phosphate fractioning from this type of adsorbents was easy obtained. However, in comparison with clays and zeolites used in our previously studies, the adsorbents used in this study are promissory due to the high content of labile phosphate that could be used to enhance plants growth.

### 3.7. Regeneration of Adsorbents

Phosphate adsorption–desorption capacities, using NaHCO_3_ (0.1 mol∙L^−^^1^ y pH 8.5) as a regenerating solution, are summarised in Table 6. The use of NaHCO_3_ for regeneration purpose was chosen due to the low adsorption capacities of the materials at pH values above 7. As discussed, phosphate adsorption mechanisms were governed by the complexation reactions to Fe–(OH)^+^ and Mn–(OH)^+^ groups. Thus, low rates of phosphate desorption were expected in this study. Phosphate from labile and residual fractions seem to be easily released from adsorbents using the NaHCO_3_ as regenerant solution. At pH 8.5, phosphate (mainly the HPO_4_^2–^ specie) could be recovered due to the reversibility of outer sphere complexes (physical adsorption). However, the chemical adsorbed phosphate complexes are non-reversible and promote the low desorption fractions (e.g., between 21 and 51%). The limited reusability of the adsorbents was determined by the stable occupancy of the Fe–(OH)^+^ and Mn–(OH)^+^ groups by phosphate. Thus, the bonding sites of the adsorbents (MC, LMC, PLMCT_3_ and SLMCT_2_) are not available for further adsorption stages. In the case of powder materials, the regenerability was lower than the densified form of the adsorbents. It is in accordance with the reusability properties of pellets and monoliths forms conventionally used for adsorption and catalytic applications.

The limited reusability of the adsorbents used in this study, enables new possibilities for final disposal of MC, LMC, PLMCT_3_ and SLMCT_2_. Phosphate adsorption–desorption processes could be performed in one cycle of operation. The MC, LMC, PLMCT_3_ and SLMCT_2_ can be finally disposal for soil amendment purposes. The high availability of labile phosphate from the saturated adsorbents used in this study (MC, LMC, PLMCT_3_ and SLMCT_2_) becomes an important source of nutrients for further agricultural applications. The provision of micro and macronutrient system (P, Fe, Mn) could be given for plants’ growth by the application of saturated MC, LMC, PLMCT_3_ and SLMCT_2_ directly to the soil.

### 3.8. Phosphate Adsorption in Continuos Mode

**Figure 10:**
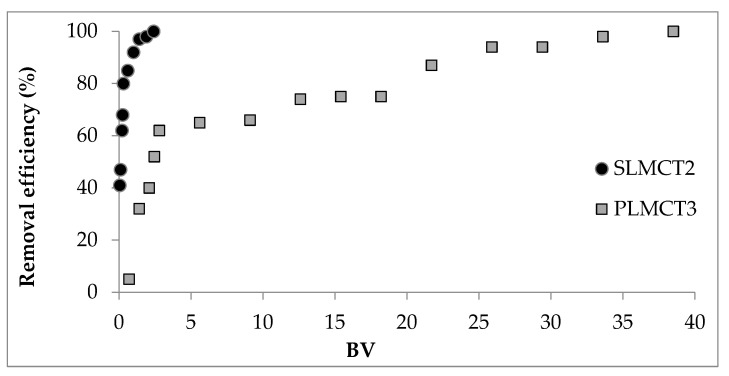
Phosphate adsorption in continuous mode onto composites SLMCT_2_ and PLMCT_3_ materials. Values obtained at C_i_: 10 mg·L^−^^1^ PO4^3^^−^, PLMCT_3_ w: 10 ± 0.3 g and SLMCT_2_ w: 10 ± 0.2 g.

The breakthrough profile of phosphate adsorption by SLMCT_2_ and PLMCT_3_ are depicted in Figure 10. Phosphate maximum sorption capacity reached at column saturation (C/C_0_ = 0.95) was 0.09 mg·g^−^^1^ PO_4_^3^^−^ for PLMCT_3_ at 35 BV. The maximum sorption capacity was reached at 0.03 mg·g^−^^1^ PO_4_^3^^−^ for SLMCT_2_ at 7 BV.

### 3.9. Implications of Phosphate Adsorption Using the Fe^3+^/Mn^2+^ Muscovite/Sodalite Composites

Phosphate adsorption capacity onto MC, LMC, SLMCT_2_ and PLMCT_3_ were negligible in comparison to other materials used for this purpose (Table 7), such as industrial adsorbents. However, the adsorption capacity values are comparable with some other adsorbents that supports metal ions. The conventionally polymeric adsorbents (e.g., resins and fibres ion exchangers) demonstrated many advantages in comparison to the inorganic materials (e.g., mechanical resistance and reusability). However, the major concern about using polymeric materials is the lack of environmentally friendly alternatives for final disposal. Some advantages and limitations are associated to the use of muscovite/zeolite composites; however, these composites provide the opportunity to work in batch (powder form) and continuous mode (pellet and monolith forms). Maybe pure metal oxide materials can provide higher adsorption capacities, but their main restriction is the particle size management problem. Thus, the use of an inorganic support (e.g., clays, zeolites) provides the opportunity of a better management of this materials.

The loaded Fe^3+^/Mn^2+^ oxy(hydroxide) muscovite/zeolite composites for phosphate recovery purposes are options for wastewater treatment operation. The application of the composites in pilot plants become possible due to the adaptability of the materials to batch (powder) and continuous mode (pellet and monoliths). The main advantage of MC, LMC, PLMCT_3_ and SLMCT_2_ over other adsorbents is their environmentally friendly alternative for final disposal. The limited reusability of the inorganic adsorbents developed in this study provides the opportunity for soil amendment application as slow nutrient release for plants growth. Thus, the loaded Fe^3+^/Mn^2+^ oxy(hydroxide) muscovite/zeolite could be used as safe phosphate-carriers from urban wastewater to soil.

## 4. Conclusions

In this study, a raw muscovite MC was used for the obtainment of muscovite/zeolite composites as the support of Fe^3+^/Mn^2+^ (oxy)hydroxide nanoparticles for phosphate adsorption. The Fe^3+^/Mn^2+^ (oxy)hydroxide nanoparticles loaded onto muscovite/sodalite powder LMC, the muscovite/analcime pellets PLMCT_3_ and the muscovite/analcime monoliths SLMCT_2_ forms were characterized and evaluated for phosphate recovery from simulated urban treated wastewater. The physicochemical characterization of the composites determined the transformation of muscovite into two new crystalline phases sodalite (LMC) and analcime (PLMCT_3_ and SLMCT_2_). The Fe^3+^ and Mn^2+^ (oxy)hydroxide incorporation into the muscovite/zeolite composites’ structure followed the occupancy of the extra-framework octahedral (outer sphere complexation) and in the framework tetrahedral sites (isomorphic substitution). The incorporation of iron and manganese (oxy)hydroxide nanoparticles onto muscovite/zeolite composites were also performed by inner sphere complexation and precipitation reactions. The powder muscovite/sodalite LMC revealed the highest phosphate adsorption capacity in comparison to the powder raw muscovite MC, pellets PLMCT_3_ and monoliths SLMCT_2_. The adsorbents used in this study developed good efficiency at the pH value of the real treated wastewater; which is an improvement in comparison to other adsorbents used for this purpose. Phosphate adsorption onto the MC, LMC, pellets PLMCT_3_ and monoliths SLMCT_2_ were promoted by physical and chemical adsorption. The formation of hydrogen bonds and monodentate and bidentate complexation governed phosphate adsorption onto the adsorbents used in this study. The kinetical data demonstrated the best fitting to the intraparticular diffusion model through two specific stages of adsorption. The fast rate of adsorption was endorsed by the physical adsorption mechanism that occurred at surface (e.g., hydrogen bonding). Then, the slow rate of chemical adsorption (e.g., chemical complexation) was promoted by the diffusion through the internal pores of the adsorbents. This explains the low phosphate adsorption capacity of pellets PLMCT_3_ and monoliths SLMCT_2_ due to the restricted access to their internal pores. Phosphate fractioning assays demonstrated that the loaded adsorbents have a high labile fraction that can be used to enhance plants growth. The limited reusability of raw muscovite MC, powder LMC, pellets PLMCT_3_ and monoliths SLMCT_2_ composites suppose a disadvantage in comparison to other adsorbents (e.g., polymeric exchangers). However, the concentrated phosphate solutions obtained from the regeneration could be used for soil amendment application, as well as the saturated adsorbents could be finally disposed for soil amendment. Thus, the use raw muscovite MC, powder LMC, pellets PLMCT_3_ and monoliths SLMCT_2_ composites in tertiary wastewater treatment stage could reduce the phosphorous contents within regulatory levels (equal 1 mg L^−1^ total phosphorous). The Fe^3+^ and Mn^2^ muscovite/zeolite composites become a new source of phosphorous for agriculture; being environmentally friendly since they did not report the release of any harmful pollutants.

## Figures and Tables

**Table 1:** Chemical composition a (weight %) and specific surface area (m^2^/g) of adsorbents.

Adsorbent Material	SiO_2_ (%)	Al_2_O_3_ (%)	MgO (%)	K_2_O (%)	CaO (%)	TiO_2_ (%)	Fe_2_O_3_ (%)	SnO_2_ (%)	MnO (%)	SA (m^2^.g^−^^1^)
MC	72 ± 0.5	12 ± 0.5	6 ± 0.2	2.5 ± 0.3	2.1 ± 0.4	0.6 ± 0.1	4 ± 0.2	0.5 ± 0.1	0.1 ± 0.1	7.0
LMC	68 ± 0.4	11 ± 0.5	1 ± 0.3	2.2 ± 0.4	0.4 ± 0.1	0.4 ± 0.1	12 ± 0.5	0.4 ± 0.1	3.5 ± 0.2	74.0
PLMCT_3_	66 ± 0.5	10 ± 0.3	1 ± 0.2	2.2 ± 0.3	0.2 ± 0.0	0.4 ± 0.1	12 ± 0.5	-	3.4 ± 0.1	1.0
SLMCT_2_	67 ± 0.5	10 ± 0.5	1 ± 0.2	2.2 ± 0.4	0.2 ± 0.0	0.4 ± 0.1	12 ± 0.4	-	3.4 ± 0.1	2.0

**Table 2:** Concentrations of ions in the exhausted loading solution determined by ICP.

Mg^2+^ (mg∙L^−^^1^)	K^+^ (mg∙L^−^^1^)	Na^+^ (mg∙L^−^^1^)	Ca^2+^ (mg∙L^−^^1^)
6	0.3	1	2

**Table 3:** Phosphate adsorption isotherm parameters for adsorbents.

Zeolite	Langmuir			Freundlich		
	$Q_{m}$ (mg·g^−1^)	$K_{L}$ (L·mg^−1^)	R^2^	$K_{F}$ (mg·g^−1^)	$\frac{1}{n}$	R^2^
MC	2.1	0.03	0.99	1.37	0.11	0.79
LMC	6.0	0.02	0.99	1.73	0.16	0.90
PLMCT_3_	0.9	0.14	0.99	0.52	0.12	0.74
SLMCT_2_	0.2	0.04	0.99	0.02	0.42	0.77

Parameters obtained at V: 25 mL, w: 0.25 g and C_i_: 10–2000 mg⋅L^−^^1^ PO_4_^3^^−^; except for SLMCT_2_ w: 10 ± 0.2 g.

**Table 4:** Kinetic parameters of phosphate adsorption for adsorbents.

Kinetic Model	Kinetic Parameter	MC	LMC	PLMCT_3_	SLMCT_2_
HPDF Film diffusion	D_f_ (m^2^·s^−^^1^)	5.4 × 10^−^^11^	3.1 × 10^−^^15^	5.2 × 10^−^^7^	2.4 × 10^−^^10^
	R^2^	0.96	0.95	0.96	0.97
HPDM Particle diffusion	D_p_ (m^2^·s^−^^1^)	2.8 × 10^−^^12^	5.6 × 10^−^^13^	3.4 × 10^−^^9^	2.9 × 10^−^^10^
	R^2^	0.95	0.97	0.96	0.97

Parameters obtained at V: 25 mL, w: 0.25 g and C_i_: 25 mg·L^−^^1^ PO_4_^3^^−^; except for SLMCT_2_, w: 10 ± 0.2 g and V: 120 mL.

**Table 5:** Fractions of phosphate bonded to the adsorbents.

Adsorbent Material	Q_e_ (mg·g^−^^1^)	LB-P		Metal-P		Alkaline-P		Residual-P	
		(mg·g^−^^1^)	%	(mg·g^−1^)	%	(mg·g^−1^)	%	(mg·g^−1^)	%
MC	0.14 ± 0.0	0.04 ± 0.0	30 ± 1	0.05 ± 0.0	39 ± 1	0.01 ± 0.0	9 ± 1	0.03 ± 0.0	22 ± 2
LMC	0.35 ± 0.0	0.12 ± 0.0	35 ± 1	0.17 ± 0.0	48 ± 1	0.02 ± 0.0	7 ± 1	0.04 ± 0.0	10 ± 2
PLMCT_3_	0.14 ± 0.0	0.05 ± 0.0	33 ± 1	0.06 ± 0.0	43 ± 1	0.01 ± 0.0	6 ± 1	0.02 ± 0.0	17 ± 2
SLMCT_2_	0.05 ± 0.0	0.016 ± 0.0	31 ± 1	0.02 ± 0.0	40 ± 1	0.004 ± 0.1	8 ± 1	0.01 ± 0.0	21 ± 2

Values obtained at V: 25 mL, w: 0.25 g and C_i_: 25 mg·L^−^^1^ PO_4_^3^^−^; except for SLMCT_2_ w: 10 ± 0.2 g.

**Table 6:** Desorption of phosphate bonded to the adsorbents.

Adsorbent Material	Q_e_ (mg·g^−^^1^)	Q_d_	Desorption %
		(mg·g^−^^1^)	
MC	0.12 ± 0.0	0.03 ± 0.0	21 ± 1
LMC	0.33 ± 0.0	0.10 ± 0.0	30 ± 1
PLMCT_3_	0.13 ± 0.0	0.05 ± 0.0	41 ± 1
SLMCT_2_	0.11 ± 0.0	0.03 ± 0.0	51 ± 1

Values obtained at V: 25 mL, w: 0.25 g and C_i_: 25 mg·L^−^^1^ PO_4_^3^^−^; except for SLMCT_2_ w: 10 ± 0.2 g.

**Table 7:** Summary of phosphate adsorption capacities of inorganic adsorbents.

Adsorbent	Description		Q_m_ (mg·g^−^^1^)	Ref.
Loaded Fe^3+^/Mn^2+^ (oxy)hydroxide nanoparticles onto muscovite/zeolite composite	Muscovite used as raw material for the synthesis of zeolite composites	MC	2.1	This study
		LMC	6.0	
		PLMCT3	0.9	
		SLMCT2	0.2	
Natural clays	Natural form	C_1_	21.4	[5]
		C_2_	20.9	
	Modified form	C_1_-Fe	38.0	
		C_2_-Fe	37.6	
Modified bentonite	Zn-containing bentonite clay		4.12	[61]
	Pillared bentonite by Fe		11.15	
Natural clays	Bentonite from Iran		0.369	[62]
	Zeolite from Iran		0.627	
	Kaolinite from Iran		0.624	
Modified bentonite	Pillared bentonite by Fe/Al		8.33	[63]
Na-Bentonites	Pillared bentonite with Al		12.7	[64]
	Pillared bentonite with Fe		11.2	
	Pillared bentonite with Fe-Al		10.5	
Synthetic zeolites	Hydrothermally synthesized	LTA-Fe	18.5	[14]
		FAU-X-Fe	17.5	
Natural zeolites	Clinoptilolite	ZN	0.6	[9]
		Z-Al	7.0	
		Z-Fe	3.4	[10]
		Z-Mn	5.6	[7]
Layered double hydroxide	Mn^2+^/Zn^2+^/Fe^3+^ Oxy(Hydroxide) layered double hydroxide	Mn^2+^/Zn^2+^/Fe^3+^/Mg-Al-LDH	82.3	[12]
Polymeric sorbent ion exchanger	Impregnated nanoparticles of hydrated ferric oxide	Lewatit FO36—HAIX	91.30	[65]
Fibrous ion exchanger	Impregnated nanoparticles of hydrated ferric oxide	FIBAN- As—FAS	161.9	[66]

## Data Availability

Not applicable.

## Supplementary Materials

The following supporting information can be downloaded at: https://www.mdpi.com/article/10.3390/nano12213848/s1, Table S1: Conventional kinetic modelling for phosphate adsorption onto muscovite composites.
