# Supplementary material for: Fe3+/Mn2+ (Oxy)Hydroxide Nanoparticles Loaded onto Muscovite/Zeolite Composites (Powder, Pellets and Monoliths): Phosphate Carriers from Urban Wastewater to Soil
Source: Nanomaterials (Basel). 2022 Oct 31;12(21):3848. doi: 10.3390/nano12213848 (PMC9654169; doi:10.3390/nano12213848)
Supplement: Supplementary file 1 [file nanomaterials-12-03848-s001.zip › nanomaterials-1972219-supplementary.pdf]

nanomaterials

MDPI

Supplementary Materials

Fe^{3+}/Mn^{2+} (Oxy)Hydroxide Nanoparticles Loaded onto Muscovite/Zeolite Composites (Powder, Pellets and Monoliths): Phosphate Carriers from Urban Wastewater to Soil

Diana Guaya [1,2,*], Luz Maza [1], Adriana Angamarca [1], Eda Mendoza [1], Luis García [1], César Valderrama [2,3] and José Luis Cortina [2,3]

1 Department of Chemistry, Universidad Técnica Particular de Loja, Loja 100107, Ecuador; lbmaza@utpl.edu.ec (L.M.); amangamarca@utpl.edu.ec (A.A.); egmendoza@utpl.edu.ec (E.M.); lvgarcia4@utpl.edu.ec (L.G.)
2 Department of Chemical Engineering, Polytechnic University of Catalonia–BarcelonaTech (UPC), 08019 Barcelona, Spain; cesar.alberto.valderrama@upc.edu (C.V.); jose.luis.cortina@upc.edu (J.L.C.)
3 Barcelona Research Center for Multiscale Science and Engineering, 08930 Barcelona, Spain
* Correspondence: deguaya@utpl.edu.ec

The kinetic data of phosphate adsorption onto clay materials were fitted to the pseudo-first order and pseudo-second order kinetic model by Equations (S1) and (S2), respectively.

$$\ln(q_e - q_t) = \ln(q_e) - k_1 t \quad \text{(S1)}$$

$$\frac{t}{q_t} = \frac{1}{k_2 q_e^2} + \frac{t}{q_e} \quad \text{(S2)}$$

where k_1 (h^{-1}) and k_2 (g mg^{-1} h^{-1}) are the kinetics constants.

The intraparticle diffusion model developed by Weber and Morris [1] also was used for describing sorption processes on the clay materials according to Equation (S3).

$$q_t = k_t\, t^{1/2} + A \quad \text{(S3)}$$

where k_t (mg g^{-1} $h^{-1/2}$) is the intraparticle diffusion rate constant and A (mg g^{-1}) is a constant that gives an indication of the thickness of the boundary layer.

Table S1. Conventional kinetic modelling for phosphate adsorption onto muscovite composites.

Kinetic model	Kinetic parameter	MC	LMC	$PLMCT_3$	$SLMCT_2$
Pseudo-first order	Q_e ($mg \cdot g^{-1}$)	0.25	1.66	0.11	0.13
	k_1 (h^{-1})	0.15	0.01	0.55	0.15
	R^2	0.95	0.96	0.96	0.98
Pseudo-second order	Q_e ($mg \cdot g^{-1}$)	0.19	1.33	0.14	0.12
	k_2 ($g \cdot mg^{-1} \cdot h^{-1}$)	0.06	0.21	0.02	0.02
	R^2	0.99	0.99	0.99	0.97
Intraparticle diffusion	k_{t1} ($mg \cdot g^{-1} \cdot h^{-1/2}$)	1.22	1.25	0.24	0.99
	R^2	0.96	0.98	0.91	0.99
	k_{t2} ($mg \cdot g^{-1} \cdot h^{-1/2}$)	0.89	0.35	0.08	0.02
	R^2	0.94	0.98	0.97	0.99
	k_{t3} ($mg \cdot g^{-1} \cdot h^{-1/2}$)	0.02	0.14	0.04	0.01
	R^2	0.98	0.95	0.91	0.99

Reference

1. Weber, W.J. and Morris, J. C. Kinetics of adsorption carbon from solutions. *Journal Sanitary Engineering Division Proceedings. American Society of Civil Engineers*, **1963**, *89*, 31–60. https://doi.org/10.1061/JSEDAI.0000430.
